# Emergence of K1 ST23 and K2 ST65 hypervirulent *klebsiella pneumoniae* as true pathogens with specific virulence genes in cryptogenic pyogenic liver abscesses Shiraz Iran

**DOI:** 10.3389/fcimb.2022.964290

**Published:** 2022-08-09

**Authors:** Maryam Sohrabi, Mahvash Alizade Naini, Alireza Rasekhi, Mana Oloomi, Farzad Moradhaseli, Abbas Ayoub, Abdollah Bazargani, Zahra Hashemizadeh, Fereshteh Shahcheraghi, Farzad Badmasti

**Affiliations:** ^1^ Department of Bacteriology, Pasteur Institute of Iran, Tehran, Iran; ^2^ Department of Internal Medicine, School of Medicine, Shiraz University of Medical Sciences, Shiraz, Iran; ^3^ Department of Radiology, Shiraz University of Medical Sciences, Shiraz, Iran; ^4^ Department of Molecular Biology, Pasteur Institute of Iran, Tehran, Iran; ^5^ Department of Bacteriology and Virology, School of Medicine, Shiraz University of Medical Sciences, Shiraz, Iran

**Keywords:** hypervirulent *Klebsiella pneumoniae*, pyogenic liver abscess, virulence, *Galleria mellonella*, sequence type

## Abstract

Hypervirulent *Klebsiella pneumoniae* (hvKp) pathotype is emerging worldwide in pyogenic liver abscesses (PLAs). However, the role of virulence factors in pathogenicity remains unclear. On the other hand, the epidemiology of PLAs in Iran is unknown. From July 2020 to April 2022, bacterial species were isolated and identified from the drainage samples of 54 patients with PLAs. *K. pneumoniae* as the most common pathogen of pyogenic liver abscesses was identified in 20 (37%) of the 54 patients. We analyzed the clinical and microbiological characteristics of *K. pneumoniae*-related pyogenic liver abscesses. Antibiotic susceptibility testes and string test were performed. 16S rRNA, antibiotic resistance, and virulence genes were determined by polymerase chain reaction amplification. Clonal relatedness of isolates was identified by multilocus sequence typing. Virulence levels were assessed in the *Galleria mellonella* larval infection model. Four hvKp isolates (K1/K2) were found to be responsible for cryptogenic PLAs, and 16 classical *K. pneumoniae* isolates (non-K1/K2) were associated with non-cryptogenic PLAs. Three capsular serotype K1 strains belonged to sequence type 23 (ST23) and one K2 strain to ST65. Meanwhile, the non-K1/K2 strains belonged to other STs. ST231 was the most common strain among the classical *K. pneumoniae* strains. Compared with the non-K1/K2 strains, capsular serotypes K1/K2 strains were less resistant to antibiotics, had positive string test results, and had more virulence genes. In *Galleria mellonella*, a concentration of 10^6^ colony-forming units of the K1 hvKp strain resulted in 100% death at 24 hours, confirming the higher virulence of the hvKp strain compared with cKp. *K. pneumoniae* isolates represented that the acquisition of any plasmid or chromosomal virulence genes contributes to pathogenicity and high prevalence in PLAs. Meanwhile, hvKp isolates with a specific genetic background were detected in cryptogenic PLAs.

## Introduction

Pyogenic liver abscess (PLA) is a rare infectious disease, but it is associated with high mortality and relapse (Heneghan et al., 2011). This pyogenic abscess is a purulent cavity in the liver parenchyma usually caused by bacteria, sometimes amoebae, and rarely fungi. These pathogens can invade the liver parenchyma either by direct route from adjacent structures, usually the bile ducts, or by hematogenous spread, often from the portal vein system (Lardière-Deguelte et al., 2015). PLA develops in the course of healthcare-associated infections, including biliary tract infections, intra-abdominal infections such as appendicitis, diverticulitis, infected gastrointestinal tumors, inflammatory bowel disease (IBD), infected liver cysts, tumors, and abdominal surgical infections. Immunosuppressive conditions including solid-organ transplantation, and diabetes mellitus (DM) have also been reported as risk factors for the development of PLA (Lardière-Deguelte et al., 2015; Mavilia et al., 2016).

PLAs are usually a polymicrobial infection caused by opportunistic pathogens of *Escherichia coli* as the most common etiologic agent, *Streptococcus* spp., anaerobes species, and classical *Klebsiella pneumoniae* (cKp) (Brook and Frazier, 1998; Serraino et al., 2018; Myeong et al., 2022). *K. pneumoniae* easily colonizes the mucosal surfaces of the human gastrointestinal tract and oropharynx. From these sites, *K. pneumoniae* isolates can invade other tissues and cause various infections, including pneumonia, urinary tract infections (UTI), sepsis, and pyogenic liver abscesses, mainly in immunocompromised patients. In *K. pneumoniae*, four major classes of virulence factors, including capsular polysaccharide, siderophores, lipopolysaccharide (LPS), and fimbriae, have been well characterized (Paczosa and Mecsas, 2016). In recent decades, the increasing prevalence of infections caused by multidrug-resistant (MDR) classical *K. pneumoniae* isolates has led to therapeutic challenges and made these isolates a public health threat (Paczosa and Mecsas, 2016). MDR isolates increase the need for new effective and safe alternatives to antibiotics such as probiotics. In addition, the routine use of antimicrobial susceptibility testing to detect the antibiotic of choice and screening of emerging MDR isolates is needed.

However, in 1986, a cryptogenic PLA complicated with metastatic septic endophthalmitis was reported in Taiwan. The abscess was caused by to a single hypervirulent *K. pneumoniae* (hvKp) microorganism (Liu et al., 1986). *Klebsiella pneumoniae-*related pyogenic liver abscesses are complicated by metastatic infections of the eye, CNS, and other sites in approximately 8-24% of cases (Siu et al., 2012). Diabetic patients are particularly predisposed to metastatic infections, including endophthalmitis and meningitis (Liu et al., 1986; Fung et al., 2002). From several studies, the mortality rate of *K. pneumoniae* liver abscesses is significantly lower than that of other pyogenic liver abscesses (4-11% versus 21-41%) (Wang et al., 1998; Yang et al., 2004; Siu et al., 2012). Today, *K. pneumoniae* liver abscess is not only considered an endemic infectious disease in Taiwan (Tsai et al., 2008), but also accounts for more than 80% of PLA cases in Southeast Asian countries (Wang et al., 1998; Chung et al., 2007; Ye et al., 2016). *K. pneumoniae*-related liver abscesses originate mainly from Southeast Asia, with increasing cases being identified in Europe, the United States, and Australia (Anstey et al., 2010; Fazili et al., 2016; Rossi et al., 2018).

It is noteworthy that mouse and *Galleria mellonella* infection models confirm increased virulence of hvKp strains compared to cKp (Fung et al., 2011; Li et al., 2020). HvKp isolates produce a hypercapsule known as the hypermucoviscous phenotype, which is detected by the string test. This hypermucoviscosity may contribute significantly to the pathogenicity of hvKp (Paczosa and Mecsas, 2016). Seventy-eight capsular serotypes have been identified in *K. pneumoniae*, and hvKp strains mostly belong to serotype K1 and, to a lesser extent, K2 (Paczosa and Mecsas, 2016). K1/K2 strains are significantly more resistant to phagocytosis and intracellular killing by neutrophils than non-K1/K2 strains, suggesting that these capsular serotypes contribute to the increased virulence of *K. pneumoniae* (Lin et al., 2004; Fung et al., 2011). Importantly, a large virulence plasmid (e.g., a pLVPK-like plasmid and pK2044) is detected in all hvKp strains. This plasmid carries virulence-associated genes, including aerobactin (*iucABCD-iutA*) and salmochelin (*iroBCDN*) siderophore gene clusters, as well as *rmpA* (regulator of mucoid phenotype) and *rmpA2* genes, which are involved in increasing capsule expression, resulting in a hypermucoviscous phenotype (Chen et al., 2004; Wu et al., 2009). These plasmids often harbor the tellurite resistance gene cluster (*terZABCDEF* and *terW*), which protects bacteria not only from tellurite toxicity but also from host defense by counteracting reactive oxygen species (ROS) produced by macrophages and neutrophils. Therefore, hvKp isolates are able to reduce tellurite to black metallic tellurium and form black colonies in tellurite-containing medium (Taylor, 1999; Franks et al., 2014; Passet and Brisse, 2015). Furthermore, variants of an integrative conjugative element of *K. pneumoniae* (ICE*Kp*) carrying the *ybt* locus encoding the siderophore yersiniabactin and its receptor are detected in approximately 18% of cKp but 90% of hvKp isolates (Paczosa and Mecsas, 2016). ICE*Kp1*, carrying the *ybt* locus as well as *iro* and *rmpA*, was identified in strain NTUH-K2044, which belongs to K1 ST23 (Wu et al., 2009). However, ICE*Kp10* carrying the *ybt* and *clb* locus (encoding the genotoxin colibactin) is more abundant in the other CC23 genomes studied (Struve et al., 2015; Lam et al., 2018a; Lam et al., 2018b). In addition, the *kfu* gene involved in iron uptake and the *allABCDRS* gene cluster associated with allantoin metabolism are frequently found in serotypes K1 (Yu et al., 2008; Luo et al., 2014). Interestingly, a study using a 694-gene core genome multilocus sequence typing scheme (cgMLST) showed that K1 strains belong to clonal complex 23 (CC23), whereas K2 strains are more genetically diverse and belong to at least four clonal complexes: CC65, CC86, CC375, and CC380 (Bialek-Davenet et al., 2014). Unlike cKp isolates, which have a high capacity to acquire antimicrobial resistance, hvKp isolates are generally not associated with antimicrobial resistance, with the exception of intrinsic resistance to ampicillin (Lee et al., 2017). However, antibiotic resistance in hvKp strains is increasing and the mortality rate in patients infected with these strains is high (Lin et al., 2018).

While these interesting results suggest a specific genetic background for *K. pneumoniae* hypervirulence, there are few data on the role of virulence factors in *K. pneumoniae* pathogenicity. On the other hand, there are no documented studies on the epidemiology of PLAs in Iran. Therefore, this cross-sectional study was designed with the aim of investigating the bacterial species of PLAs in Shiraz, Iran. We also focused on the clinical and microbiological characteristics of *K. pneumoniae*-related pyogenic liver abscesses in order to determine the role of virulence factors in *K. pneumoniae* pathogenicity in clinical settings.

## Materials and methods

### Patients study

We performed this cross-sectional study in three clinical settings including Abu Ali Sina Hospital as the main transplant hospital in Iran with a history of more than 6000 liver transplants in the last 25 years, Namazi Hospital as the main tertiary teaching hospital, and Fara Parto Medical Imaging and Interventional Radiology Center in Shiraz. Drainage samples of pyogenic liver abscess were collected aseptically from all patients diagnosed with liver abscess. The diagnosis of liver abscess was based on the typical clinical presentation (fever, abdominal pain), biological abnormalities (leukocytosis, abnormal liver function tests), and imaging of liver abscesses by computed tomography, ultrasonography, or magnetic resonance imaging (Lardière-Deguelte et al., 2015; Mavilia et al., 2016). Documented cases with positive bacterial culture of PLA were included in the study. The history, physical examination, and medical records of all patients were reexamined by a gastroenterologist for possible sources of infection or underlying diseases, such as biliary tract, peritonitis, and diverticulitis or other intra-abdominal infections, recent abdominal surgery, transplantation, cancer, inflammatory bowel disease, and diabetes mellitus. Based on the underlying disease, cases with *K. pneumoniae* liver abscess were divided into two groups: cryptogenic and non-cryptogenic liver abscesses. The clinical, radiological, and microbiological characteristics of the patients and the outcomes of both groups were statistically analyzed.The studies involving human participants were reviewed and approved by the ethics committee of the Pasteur Institute of Iran (reference number: IR.PII. REC.1399.065).The patients provided their written informed consent to participate in this study.

### Key definitions

Classical *K. pneumoniae* (cKp) is an opportunistic pathogen causing infections primarily in immunocompromised individuals with underlying diseases, whereas hypervirulent *K. pneumoniae* (hvKp) causes cryptogenic invasive infections in healthy individuals (Paczosa and Mecsas, 2016). A cryptogenic liver abscess is defined as PLA in the absence of underlying disease (Lardière-Deguelte et al., 2015; Mavilia et al., 2016; Rossi et al., 2018), and a non-cryptogenic as PLA in the presence of underlying diseases such as biliary tract infections, appendicitis, diverticulitis, infected gastrointestinal tumors, inflammatory bowel disease, infected liver cysts and tumors, abdominal surgical infections, transplantation, cancer, and diabetes mellitus. Monomicrobial infection is defined when only *K. pneumoniae* is detected alone, and polymicrobial when *K. pneumoniae* is detected together with other pathogens in pus culture and Gram stain (Rossi et al., 2018). Diabetes mellitus is defined as random plasma glucose ≥ 200 mg/dl, fasting plasma glucose ≥ 126 mg/dl, or 2-hour plasma glucose ≥ 200 mg/dl (Organization, 2018).

### Bacterial isolation and identification

Bacterial species of PLA were isolated from drainage samples and identified by standard biochemical tests, including indole test, methyl red test (MR), Voges-Proskauer test (VP), citrate utilization test, triple sugar iron test (TSI), urease test, and bile esculin test. The pus sample was cultured on blood agar (HiMedia, India) and McConkey agar (HiMedia, India) using the Streak technique. For isolation of anaerobes, the blood medium was incubated anaerobically using the Anoxomat system (Mart Microbiology B.V., The Netherlands). Lactose-fermenting pink mucoid colonies on McConkey medium (a selective and differential medium) with positive catalase and negative oxidase tests and Gram-negative bacilli morphology in Gram stain were suspected to be *K. pneumoniae*. To determine whether the culture was mixed with other bacterial species, the shape and size of the colonies on the blood medium (an enriched medium) and the Gram stain, catalase and oxidase tests were checked simultaneously, and a bile esculin test was performed. Biochemical tests were then performed. The colonies that made the TSI medium completely acidic (yellow color) and by adding reagents of 5% alpha-naphthol and 40% KOH and MR reagent (Ibresco, Iran) became VP positive (red color) and MR negative (yellow color), respectively. At the same time, they were urease positive (pink color), citrate positive (blue color) and immobilized in SIM medium and became negative-indol by adding Kovac′s reagent, they were identified as *K. pneumoniae* (Mahon et al., 2018). Each *K. pneumoniae* isolate was confirmed as *K. pneumoniae* by PCR and DNA sequencing of the 16S rRNA gene (Juretschko et al., 2002). *K. pneumoniae* isolates were stored in Tryptic Soy Broth (TSB) containing 30% glycerol at minus 70°C for further characterization.

### Antimicrobial susceptibility testing

Antimicrobial susceptibility of *K. pneumoniae* isolates was determined by the Kirby-Bauer disk diffusion method on Mueller-Hinton agar (HiMedia, India) according to Clinical Laboratory Standards Institute guidelines (CLSI 2020-M100-S30) (Weinstein et al., 2020). A panel of 14 antimicrobial agents was used, including *β*-lactam antibiotics (i.e. penicillins, third- and fourth-generation cephalosporins, monobactams, and carbapenems) ampicillin (AMP), ceftriaxone (CRO), cefotaxime (CTX), ceftazidime (CAZ), cefepime (CPM), aztreonam (ATM), imipenem (IMI), meropenem (MEM), ertapenem (ERP); aminoglycoside antibiotics gentamicin (CN), amikacin (AK); fluoroquinolone antibiotic ciprofloxacin (CIP); and *β*-lactam antibiotics in combination with the *β*-lactamase inhibitor cefotaxime-clavulanate (CTX-CV), ceftazidime-clavulanate (CAZ-CV). According to the antibiotic resistance profile, isolates resistant to at least one agent in three or more antimicrobial categories were defined as multidrug-resistant (MDR) (Magiorakos et al., 2012). Minimum inhibitory concentrations (MICs) of imipenem and ceftazidime were determined by the broth microdilution method according to the CLSI recommendation. Isolates showing resistance to one or more third-generation cephalosporins (e.g., ceftriaxone, cefotaxime, and ceftazidime) or resistance to one or more carbapenems (e.g., imipenem, meropenem, and ertapenem) were selected for detection of extended-spectrum-*β*-lactamase (ESBL) and carbapenemase production by phenotypic confirmatory disk diffusion test (PCDDT) and modified carbapenem inactivation method (mCIM) according to CLSI guidelines. *E. coli* ATCC 25922 (antibiotic sensitive), *K. pneumoniae* ATCC 700603 (ESBL producer), and *K. pneumoniae* ATCC 1705 (carbapenemase producer) were used as quality control strains for antimicrobial susceptibility testing.

### String test and tellurite resistance

The hypermucoviscous (HV) phenotype of *K. pneumoniae* isolates was studied with the string test. A positive string test is defined as the formation of a viscous string >5 mm in length when colonies grown overnight on a blood agar plate at 37°C are stretched with a bacteriological loop (Lee et al., 2017). The tellurite resistance phenotype was studied in *K. pneumoniae* isolates. Isolates that form black colonies on the tellurite-containing selective medium are resistant to tellurite. To perform this assay, 0.1 g of potassium tellurite powder was dissolved in 10 ml of sterile distilled water and filtered with a membrane filter of pore size 0.45 μm. Then 300 μl of this potassium tellurite solution was added to 100 ml of Mueller–Hinton agar medium, which was autoclaved and cooled to 45–50°C. Finally, we poured it into sterile plates and examined the colonies after overnight incubation at 37°C (Sanikhani et al., 2021).

### Polymerase chain reaction

Genomic DNA of *K. pneumoniae* isolates was extracted using the Tissue Genomic DNA Extraction Mini Kit (FAVORGEN Biotech Corporation, Taiwan). Subsequently, specific genes for capsular serotypes K1 and K2 and virulence genes including *rmpA*, *rmpA2*, *iutA*, *iucA*, *iroB*, *kfu*, *allS*, *entB*, *ybtS*, *clbA*, as well as the *β*-lactamase genes such as *bla*_TEM_ *, bla*_SHV_ *, bla*_CTX-M_ *, bla*_KPC_ *, bla*_NDM_, and *bla*_OXA-48_ were identified by PCR with the specific primers (**Table 1**). Positive PCR products were submitted for sequencing and subsequently searched in the GenBank database using the tool BLAST (http://www.ncbi.nlm.nih.gov/blast/).

**Table 1:** Specific pairs of primers were used to amplify the target genes of *K. pneumoniae* in this study.

Target gene	Primer	Sequence (5′–3′)	Product size (bp)	Annealing temperature (°C)	Reference
Capsular type K1	magA-F magA-R	GGTGCTCTTTACATCATTGC GCAATGGCCATTTGCGTTAG	12082	51	(Compain et al., 2014)
Capsular type K2	K2wzy-F K2wzy-R	GGATTATGACAGCCTCTCCT CGACTTGGTCCCAACAGTTT	908	50	(Fang et al., 2007)
*rmpA*	rmpA-F rmpA-R	GAGTATTGGTTGACAGCAGGAT AGCCGTGGATAATGGTTTACA	250	53	(Sanikhani et al., 2021)
*rmpA2*	rmpA2-F rmpA2-R	ACGTATGAAGGCTCGATGGATA CCTCCTGGAGAGTAAGCATTGT	354	60	This study
Aerobactin	iutA-F iutA-R	GCCGCTAGGTTGGTGATGT CTCTGGTCGTGCTGGTTGA	949	61	Sanikhani et al., 2021)
Aerobactin	iucA-F iucA-R	AATCAATGGCTATTCCCGCTG CGCTTCACTTCTTTCACTGACAG	239	61	(Russo et al., 2018)
Salmochelin	iroB-F iroB-R	GTGTTGGATTCCGCCAGTGA TTCCGCCGCTACCTCTTC	366	61	(Sanikhani et al., 2021)
Enterobactin	entB-F entB-R	GAGCAGAGCGATGAAGAC CCGAATCCAGACCGTAGT	487	62	This study
Yersiniabactin	ybtS-F ybtS-R	GACGGAAACAGCACGGTAAA GAGCATAATAAGGCGAAAGA	242	60	(Compain et al., 2014)
*pks(clbA)*	clbA-F clbA-R	CCAGTAGAGATAACTTCCTTCA GCTGATAGTCGTGGTGATAA	660	56	This study
*kfu*	kfu-F kfu-R	ATAGTAGGCGAGCACCGAGA AGAACCTTCCTCGCTGAAC	520	60	(Yu et al., 2008)
Allantoin	allS-F allS-R	CCGAAACATTACGCACCTTT ATCACGAAGAGCCAGGTCAC	508	60	(Yu et al., 2008)
16S rRNA	16S rRNA-F 16S rRNA-R	AGAGTTTGATYMTGGCTC CAKAAAGGAGGTGATCC	1536	50	(Juretschko et al., 2002)
*bla*_TEM_	TEM-F TEM-R	GAGTATTCAACATTTCCGTGTC TAATCAGTGAGGCACCTATCTC	800	54	(Ahmed et al., 2014)
*bla*_SHV_	SHV-F SHV-R	AAGATCCACTATCGCCAGCAG ATTCAGTTCCGTTTCCCAGCGG	200	60	(Ahmed et al., 2014)
*bla*_CTX-M_	CTX_M_-F CTX_M_-R	CGCTTTGCGATGTGCAG ACCGCGATATCGTTGGT	550	55	Sanikhani et al., 2021)
*bla*_KPC_	KPC-F KPC-R	CGTCTAGTTCTGCTGTCTTG CTTGTCATCCTTGTTAGGCG	798	55	(Abhari et al., 2019)
*bla*_NDM-1_	NDM-F NDM-R	GGTTTGGCGATCTGGTTTTC CGGAATGGCTCATCACGATC	621	54	(Abhari et al., 2019)
*bla*_OXA−48_	OXA-F OXA-R	GCGTGGTTAAGGATGAACAC CATCAAGTTCAACCCAACCG	745	60	(Ahmed et al., 2014)

### Multilocus sequence typing

MLST was performed by amplification and sequencing of seven housekeeping genes of *K. pneumoniae* (*gapA, mdh, phoE, tonB, infB, pgi*, and *rpoB*) according to the protocols available on the Pasteur Institute MLST website for *K. pneumoniae* (https://bigsdb.pasteur.fr/klebsiella/).

### Assessment of virulence in *galleria mellonella* infection model

Virulence levels were assessed in *Galleria mellonella* larva, which has been reported to be a suitable *in vivo* model for *K. pneumoniae* infection (Insua et al., 2013; Tsai et al., 2016). According to the mode of acquisition of liver abscess and genotypic characteristics, a serotype K1 responsible for cryptogenic PLA and a serotype non-K1/K2 responsible for non-cryptogenic PLA were selected to further evaluate the level of virulence. Larvae of 2 to 3 cm and weighing approximately 250 to 350 mg, were prepared by the Biological Control Research Department, Plant Pathology Research Institute, Tehran, Iran, and maintained at 21°C in the dark with an unrestricted diet. Overnight cultures of *K. pneumoniae* were washed with 10 mM phosphate-buffered saline (PBS; pH 6.5) and adjusted for serial dilutions containing 10^4^ to 10^7^ CFU/ml with PBS. *G. mellonella* larvae were injected with the serial dilutions as previously described, placed in Petri dishes, and stored at 37°C in the dark. The number of dead larvae, taking into account melanization and immobility, was recorded 24, 48, and 72 hours after injection (Insua et al., 2013; Gu et al., 2018). Experiments were performed in triplicate.

### Statistical analysis

Statistical analysis of the data was performed with SPSS Statistics Version 22.0 Release 2013 (IBM SPSS Statistics for Windows, Armonk, NY, USA). Fisher’s exact test was used to compare the clinical and microbiological data of patients with cryptogenic and non-cryptogenic *K. pneumoniae* liver abscesses. The statistical correlation test, Pearson’s correlation coefficient, was determined at the two-way significance level between phenotypic and genotypic multidrug resistance. P values <0.05 were considered statistically significant.

## Results

### Clinical characteristics

#### Patients

**Figure 1:**
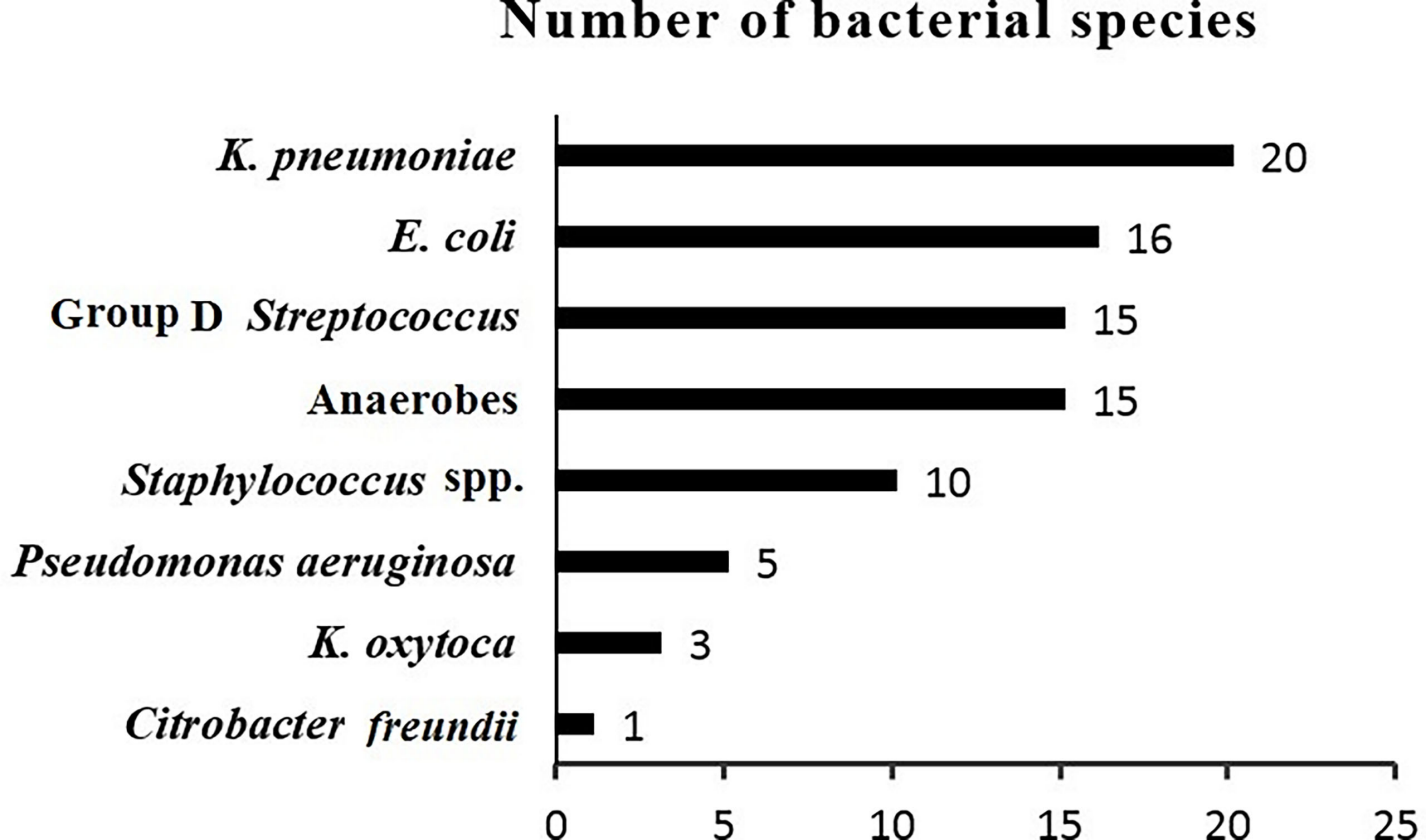
Bacterial species from patients with pyogenic liver abscesses.

During the study period from July 2020 to April 2022, a total of 76 inpatients and outpatients were diagnosed with PLA. After pus culture, 54 patients with positive bacterial cultures were included in our study. The bacteria were isolated and identified. *K. pneumoniae* was the most frequently isolated species and was detected in 20 (37%) of the 54 patients. In contrast, 34 (63%) of the 54 patients had non-*K. pneumoniae* liver abscess. The bacterial species isolated from patients with PLAs are demonstrated in **Figure 1**.

Of the patients with *K. pneumoniae* liver abscesses (20 cases), 4 (20%) had cryptogenic liver abscesses and 16 cases (80%) had non-cryptogenic liver abscesses. The male-to-female ratio for cryptogenic and non-cryptogenic cases was 3/1 and 8/8, respectively. The mean age of patients with cryptogenic and non-cryptogenic liver abscesses was 45 and 48 years, respectively.

#### Clinical course:

##### A: Clinical presentation

The most common clinical symptoms were fever (100% in both groups) and abdominal pain 75% in cryptogenic and 69% in non-cryptogenic cases (P value=0.657).

##### B: Underlying diseases

Of the patients with non-cryptogenic liver abscesses (no: 16), eight patients had liver transplantation due to liver cirrhosis (two with Wilson’s disease, three due to hepatitis B, two due to biliary tract obstruction, and one with autoimmune biliary cirrhosis), one had kidney transplantation, two patients with biliary tract stenosis and infection, two patients with pancreatic cancer, one with gastrointestinal stromal tumor (GIST), one after surgery for a liver hydatid cyst, and one with diabetes as the only underlying disease with a plasma glucose level in the range of 200-240 mg/dl.

Before the onset of pyogenic liver abscesses, all four patients in the cryptogenic liver abscess group were in good health and without underlying disease, but one had a history of liver abscesses with 5 relapses. Of the four patients with cryptogenic abscess, three had a single K1 *K. pneumoniae* abscess in the right lobe and one had a K2 *K. pneumoniae* abscess in the left lobe, all with comparable size (nearly 300 cm ^3^). The patient with a history of pyogenic liver abscess had a single relapse after treatment. Compared with the cryptogenic group, immunosuppressive conditions were the most common risk factors (81%, 13/16) among patients in the non-cryptogenic liver abscess group. On the other hand, seven relapses (44%) and 10 deaths (63%) were observed in 16 patients with non-cryptogenic *K. pneumoniae* liver abscess. The high mortality rate of patients in the non-cryptogenic liver abscess group was significantly associated with immunosuppressive conditions (P value=0.029)

##### C: Para-clinical information

#### White blood cells

Biological parameters reflect a chronic inflammatory state in cases with PLA. Leukocytosis (up to 23200) was detected in 75% of cases with cryptogenic liver abscess and in 50% of non-cryptogenic cases (P value=0.375).

#### Estimated sedimentation rate, C-reactive protein

CRP and ESR were elevated in all cases (100%), and the mean levels of these parameters were 85 mg/L and 66 mm/h, respectively.

#### Liver function tests

Alkaline phosphatase (ALP), Serum glutamic pyruvate transaminase (SGPT or ALT), Serum glutamic oxaloacetic transaminase (SGOT or AST), and Total bilirubin (TB):

Elevated ALP levels up to 697 U/L were observed in 75% of cases with cryptogenic liver abscess and in 100% of non-cryptogenic cases. Elevated levels of ALT and AST (up to 425 and 684 U/L, respectively) were observed in 100% of cases with cryptogenic liver abscess and in 75% of non-cryptogenic cases. Mild elevation (up to 6.95 mg/dl) in TB levels was observed in 75% of cryptogenic and 88% of non-cryptogenic cases.

##### D: Radiological findings

Of them, cryptogenic *K. pneumoniae* liver abscesses were frequently solitary, and three of them were localized in the right lobe of the liver (3/4, 75%), and a single abscess in the left lobe of the liver (25%), whereas nine abscesses of non-cryptogenic *K. pneumoniae* liver abscesses were localized in the right lobe of the liver (56%), six (38%) in both the left and right lobes, and only one case (6%) in the left lobe.

##### E: Size of abscesses

The mean size of abscesses in patients with non-cryptogenic liver abscesses was less than cryptogenic liver abscesses (nearly 120 and 300 cm^3^ respectively).

### Microbiological data

#### Microbial type of infection

All four cryptogenic *K. pneumoniae* liver abscesses were monomicrobial infections, whereas only 2/16 non-cryptogenic *K. pneumoniae* liver abscesses were monomicrobial and the rest were polymicrobial (P value=0.03). We identified the characteristics of 20 K*. pneumoniae* isolates from cryptogenic and non-cryptogenic liver abscesses (**Table 2**). The clinical and microbiological data confirmed that all four *K. pneumoniae* isolates from cryptogenic pyogenic liver abscesses were hypervirulent *K. pneumoniae*.

**Table 2:** Characteristics of 20 K*. pneumoniae* isolates from 4 cryptogenic liver abscesses and 16 non-cryptogenic liver abscesses.

Mode of abscess acquisition and microbial type	Chromosomal virulence genes						Plasmid virulence genes					Srting test result	Sequence type	Patho type
	K1/ K2	*entB*	*ybtS*	*clbA*	*kfu*	*allS*	*rmpA*	*rmpA2*	*iucA*	*iutA*	*iroB*
**Cryptogenic**														
Monomicrobial	K1	+	+	+	+	+	+	+	+	+	+	+	ST23	hvKp
Monomicrobial	K1	+	+	+	+	+	+	+	+	+	+	+	ST23	hvKp
Monomicrobial	K1	+	+	+	+	+	+	+	+	+	+	+	ST23	hvKp
Monomicrobial	K2	+	+	–	–	–	+	+	+	+	+	+	ST65	hvKp
**Non-cryptogenic**														
Monomicrobial	–	+	+	–	–	–	–	–	–	–	–	–	ST54	cKp
Monomicrobial	–	+	–	–	+	–	–	–	–	–	–	–	ST4023	cKp
Polymicrobial	–	+	–	–	–	–	–	–	–	–	–	–	ND	cKp
Polymicrobial	–	+	–	–	–	–	–	–	–	–	–	–	ST2935	cKp
Polymicrobial	–	+	+	–	–	–	–	–	–	–	–	–	ND	cKp
Polymicrobial	–	+	+	–	–	–	–	–	–	–	–	–	ST16	cKp
Polymicrobial	–	+	–	–	–	–	–	–	–	–	–	–	ND	cKp
Polymicrobial	–	+	+	–	+	–	–	–	–	–	–	–	ST231	cKp
Polymicrobial	–	+	+	–	–	–	–	–	–	–	–	–	ST628	cKp
Polymicrobial	–	+	+	–	–	–	–	–	–	–	–	–	ST36	cKp
Polymicrobial	–	+	+	–	–	–	–	–	–	–	–	–	ND	cKp
Polymicrobial	–	+	+	–	–	–	–	–	–	–	–	–	ST377	cKp
Polymicrobial	–	+	–	–	–	–	–	–	–	–	–	–	ND	cKp
Polymicrobial	–	+	–	–	–	–	–	–	–	–	–	–	ND	cKp
Polymicrobial	–	+	+	–	+	–	–	–	–	–	–	–	ST231	cKp
Polymicrobial	–	+	+	–	+	–	–	–	–	–	–	–	ST231	cKp

^*^+, positive; -, negative. ND, not determined.

### Phenotypic characteristics of K. pneumoniae isolates:

#### String test and tellurite resistance

**Figure 2:**
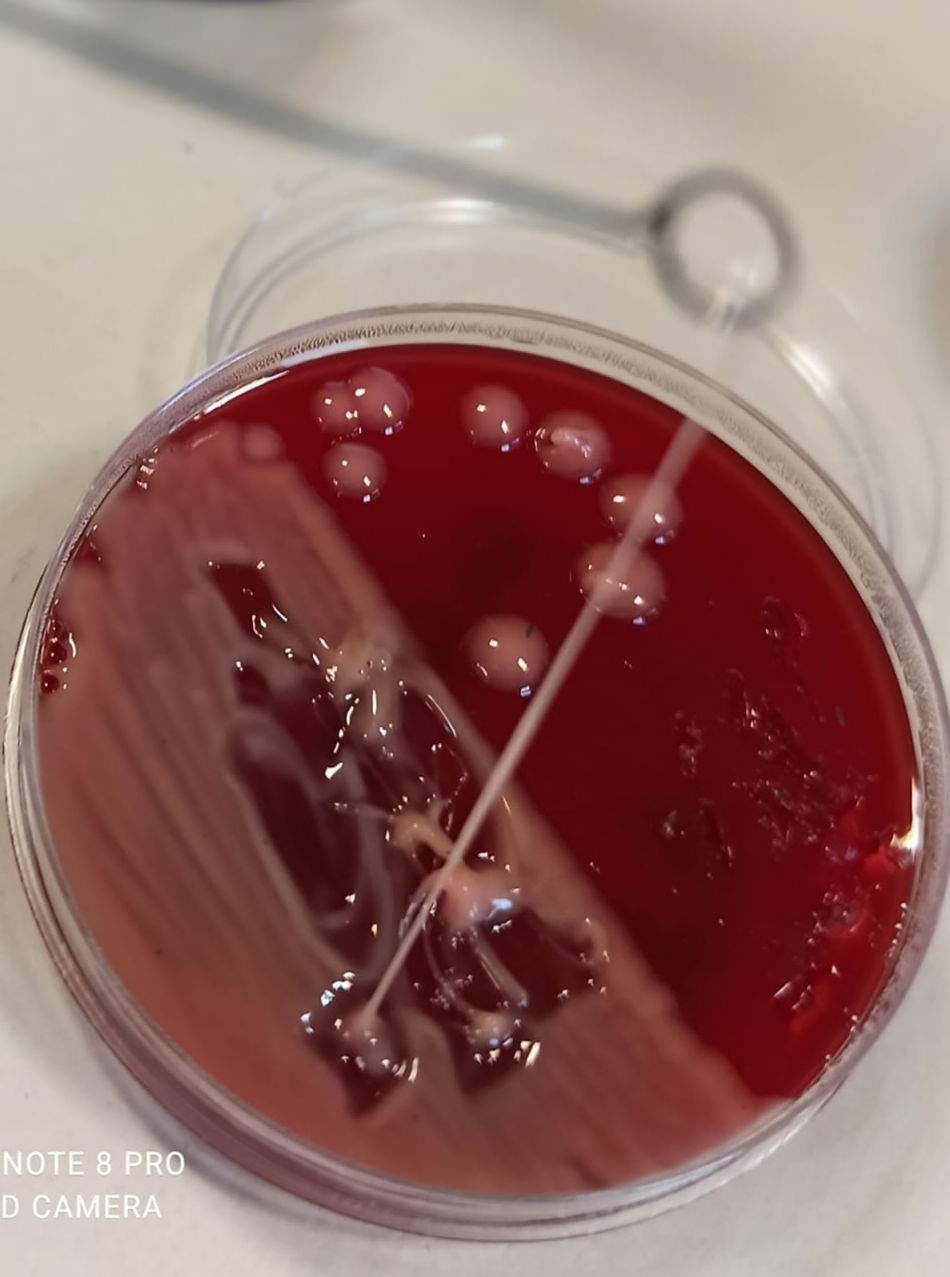
Positive string test of capsular serotypes K1/K2 of *K. pneumoniae*.

Four *K. pneumoniae* isolates from cryptogenic PLAs belonged to capsular serotypes K1/K2 and 16 K*. pneumoniae* isolates from non-cryptogenic PLAs belonged to capsular serotypes non-K1/K2. On the blood agar plate, capsular serotypes K1/K2 formed colonies with a distinct hypermucoviscous phenotype, which was not observed in non-K1/K2.The results of the string test were positive only for all four capsular serotypes K1/K2 of *K. pneumoniae*, each of which formed a viscous string longer than 20 mm (**Figure 2**). When the tellurite resistance phenotype was examined, 80% (16/20) of *K. pneumoniae* isolates, including all four capsular serotypes K1/K2, and 12 of the 16 non-K1/K2 strains were able to grow on the tellurite-containing Mueller-Hinton medium and formed black colonies. These isolates were resistant to tellurite and there was no significant difference between K1/K2 and non-K1/K2 according to this phenotype (P value=0.376).

### Genotypic characteristics of K. pneumoniae isolates:

#### Virulence genes and clonal relatedness

The *entB* gene was detected in all 20 K*. pneumoniae* isolates in both groups. Virulence genes, including *rmpA, rmpA2, iucA, iutA*, and *iroB*, were detected only in capsular serotypes K1/K2, which were responsible for cryptogenic pyogenic liver abscesses. Three chromosomal virulence genes, *kfu, allS*, and *clbA*, were detected in serotype K1 but not in K2. Comparing of K1/K2 with serotypes non-K1/K2 from non-cryptogenic pyogenic liver abscesses represented that the *ybtS* gene was detected in all (100%) capsular serotypes K1/K2 and 10 (63%) of 16 non-K1/K2 strains. The *kfu* gene was detected in capsular serotype K1 and in 4 (25%) of 16 non-K1/K2 strains. The *allS* and *clbA* genes were detected only in capsular serotype K1. The serotypes K1 belonged to ST23, whereas the serotype K2 belonged to ST65. Meanwhile, non-K1/K2 strains belonged to other STs (ST54, 4023, 2935, 16, 231, 628, 36, and 377). ST231 was the most common strain among the non-K1/K2 strains (**Table 2**). Genotyping of *K. pneumoniae* revealed the heterogeneity of *K. pneumoniae* isolates. Meanwhile, it showed the clonal diffusion of capsular serotype K1 belonging to ST23.

### Antimicrobial resistance

As expected, only the ampicillin resistance phenotype mediated by the chromosomal gene *bla*_SHV_ was identified in *K. pneumoniae* isolates from cryptogenic PLAs. In contrast, significant antibiotic resistance was observed in *K. pneumoniae* isolates from non-cryptogenic PLAs. All phenotypic and genotypic tests represented that in multidrug-resistant (MDR) *K. pneumoniae* isolates, the production of ESBL and carbapenemase *β*-lactamases was the major mechanism of antibiotic resistance. According to the antibiotic resistance profile, 8 (50%) of 16 isolates were resistant to at least three antimicrobial categories and were defined as MDR (Magiorakos et al., 2012). Among the MDR isolates, the antibiotic resistance rate was 100% (8/8) for ampicillin, ceftriaxone, cefotaxime, ceftazidime, cefepime, aztreonam, ciprofloxacin, 88% (7/8) for imipenem, meropenem, ertapenem, gentamicin, and 75% (6/8) for amikacin. Subsequently, the MICs of imipenem and ceftazidime were determined by the broth microdilution method. All 8 MDR isolates had a MIC ≥ 16 for ceftazidime: one isolate had MIC=16, one isolate had MIC=32, one isolate had MIC=64, and five isolates had MIC=128. For imipenem, 7 of the 8 MDR isolates had a MIC ≥ 4: for one isolate MIC=4, four isolates MIC=16, two isolates MIC=32. Of the MDR isolates, 88% (7/8) were co-producers of ESBLs and carbapenemase, and one isolate was ESBLs producer (**Table 3**). The results of phenotypic PCDDT and mCIM testing of the MDR isolates were fully correlated to their antibiotic resistance genotype (r = 1, P value< 0.01), and the most common genotype among the MDR isolates was *bla*_OXA-48_ (88%, 7/8), *bla*_CTX-M_ (100%, 8/8), *bla*_SHV_ (100%, 8/8), and *bla*_TEM_ (75%, 6/8) (**Table 3**).

**Table 3:** Antibiotic class, antibiotic resistance profile, antibiotic resistance gene, and susceptibility profile in 20 K*. pneumoniae* isolates from cryptogenic and non-cryptogenic liver abscesses.

Capsular type	Antibiotic class	Antibiotic resistance profile	Antibiotic resistance gene	Susceptibility profile	Sequence type
K1 K1 K1 K2	*β*-lactam *β*-lactam *β*-lactam *β*-lactam	APM APM APM APM	*bla*_SHV_ *bla*_SHV_ *bla*_SHV_ *bla*_SHV_	Susceptible Susceptible Susceptible Susceptible	ST23 ST23 ST23 ST65
Non-K1/K2	*β*-lactam Aminoglycoside, Fluoroquinolone	AMP, CRO, CTX, CAZ, CPM, ATM, IMI, MEM, ERP, CN, AK, CIP	*bla*_SHV_ *, bla*_TEM_ *, bla*_CTX-M_ *, bla*_OXA-48_	MDR	ST54
Non-K1/K2	*β*-lactam, Fluoroquinolone	AMP, CRO, CTX, CAZ, CPM, ATM, CIP	*bla*_SHV_ *, bla*_TEM_ *, bla*_CTX-M_	MDR	ST4023
Non-K1/K2	*β-*lactam	AMP	*bla*_SHV_	Susceptible	ND
Non-K1/K2	*β-*lactam	AMP	*bla*_SHV_	Susceptible	ST2935
Non-K1/K2	*β-*lactam, Aminoglycoside, Fluoroquinolone	AMP, CRO, CTX, CAZ, CPM, ATM, IMI, MEM, ERP, CN, AK, CIP	*bla*_SHV,_ *bla*_CTX-M_ *, bla*_OXA-48_	MDR	ND
Non-K1/K2	*β*-lactam, Aminoglycoside, Fluoroquinolone	AMP, CRO, CTX, CAZ, CPM, ATM, IMI, MEM, ERP, CN, AK, CIP	*bla*_SHV_, *bla*_CTX-M_, *bla*_OXA-48_	MDR	ST16
Non-K1/K2	*β*-lactam	AMP	*bla*_SHV_	Susceptible	ND
Non-K1/K2	*β*-lactam, Aminoglycoside, Fluoroquinolone	AMP, CRO, CTX, CAZ, CPM, ATM, IMI, MEM, ERP, CN, AK, CIP	*bla*_SHV_ *, bla*_TEM_ *, bla*_CTX-M_ *, bla*_OXA-48_	MDR	ST231
Non-K1/K2	*β-*lactam	AMP	*bla*_SHV_ *, bla*_TEM_	Susceptible	ST628
Non-K1/K2	*β-*lactam	AMP	*bla*_SHV_ *, bla*_TEM_	Susceptible	ST36
Non-K1/K2	*β-*lactam	AMP	*bla*_SHV_	Susceptible	ND
Non-K1/K2	*β-*lactam, Aminoglycoside, Fluoroquinolone	AMP, CRO, CTX, CAZ, CPM, ATM, IMI, MEM, ERP, CN, CIP	*bla*_SHV_ *, bla*_TEM_ *, bla*_CTX-M_ *, bla*_OXA-48_	MDR	ST377
Non-K1/K2	*β-*lactam	AMP	*bla*_SHV_	Susceptible	ND
Non-K1/K2	*β-*lactam	AMP	*bla*_SHV,_ *bla*_TEM_	Susceptible	ND
Non-K1/K2	*β-*lactam, Aminoglycoside, Fluoroquinolone	AMP, CRO, CTX, CAZ, CPM, ATM, IMI, MEM, ERP, CN, AK, CIP	*bla*_SHV_ *, bla*_TEM_ *, bla*_CTX-M_ *, bla*_OXA-48_	MDR	ST231
Non-K1/K2	*β-*lactam, Aminoglycoside, Fluoroquinolone	AMP, CRO, CTX, CAZ, CPM, ATM, IMI, MEM, ERP, CN, AK, CIP	*bla*_SHV_ *, bla*_TEM_ *, bla*_CTX-M_ *, bla*_OXA-48_	MDR	ST231

*ND, not determined.

### *G. mellonella* infection model

**Figure 3:**
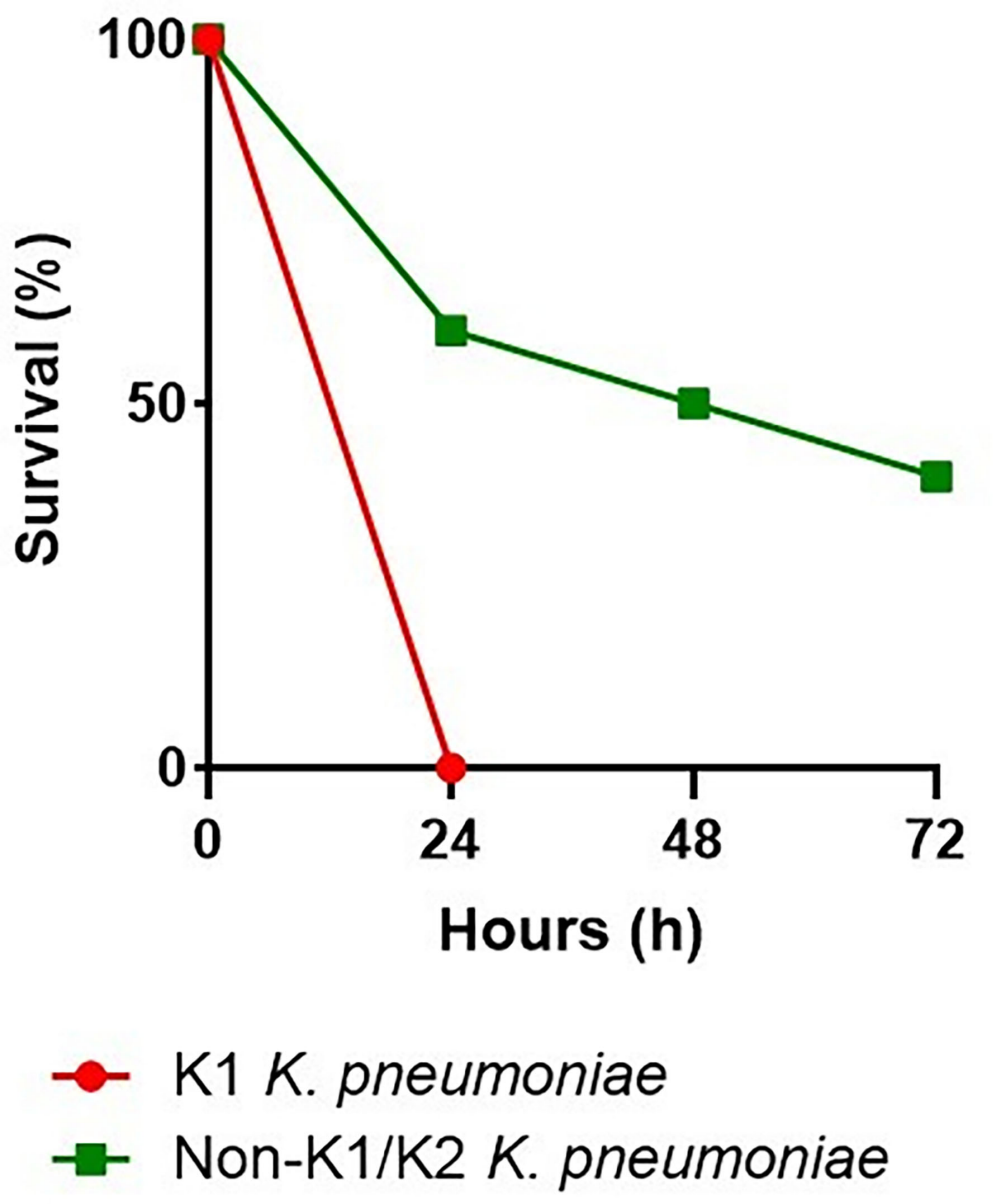
Virulence levels of the K1 *K. pneumoniae* strain and the non-K1/K2 *K. pneumoniae* strain in the *G. mellonella* infection model. In *G. mellonella*, the survival rate after an injection of 10^6^ CFU with the serotype K1 was 0% at 24 hours, compared with the serotype non-K1/K2, which was 60%, 50%, and 40% at 24, 48, and 72 hours, respectively.

We infected *G. mellonella* larvae to compare the virulence of two strains of *K. pneumoniae*. One of serotype K1, responsible for cryptogenic PLA, and the other of serotype non-K1/K2, responsible for non-cryptogenic PLA. Mortality of infected larvae was dose- and strain-dependent. In addition, non-K1/K2 *K. pneumoniae* triggered time-dependent larval death, which was less common with K1 *K. pneumoniae*. Concentrations of 10^7^ and 10^6^ CFU of serotype K1 resulted in 100% death 24 h after injection, compared with serotype non-K1/K2, in which injection of 10^7^ CFU resulted in 50% death and 70% death at 24 and 72 h, respectively. Injection of 10^6^ CFU resulted in 40% and 60% death at 24 and 72 h, respectively. Serotype K1 at concentrations of 10^5^ and 10^4^ CFU resulted in 100% death at 48 hours, compared with serotype non-K1/K2, which resulted in 30% death at 72 hours at a concentration of 10^5^ CFU and did not result in death at a concentration of 10^4^ CFU. The effect of 10^6^ CFU of each of these two strains of *K. pneumoniae* was demonstrated by the survival of *G. mellonella* larvae (**Figure 3**).

## Discussion

Pyogenic liver abscess, once associated with a mortality rate of 80%, now has a better prognosis thanks to improved diagnostic, therapeutic, and supportive care (Heneghan et al., 2011). More recently, the mortality rate has been 21-41% (Wang et al., 1998; Yang et al., 2004). Some underlying diseases such as intra-abdominal infections, immunodeficiency, and diabetes mellitus are the main predisposing factors for the development of PLA (Lardière-Deguelte et al., 2015; Mavilia et al., 2016). Although most PLAs are polymicrobial and opportunistic intestinal pathogens, particularly *E. coli* and anaerobes species, are the most important pathogens identified (Myeong et al., 2022), monomicrobial infection with hvKp has been the most important causative agent of primary or cryptogenic liver abscesses in Asia over the past three decades. The *K. pneumoniae* isolates that cause PLAs have increased numbers of virulence factors compared with other *K. pneumoniae* isolates (Ye et al., 2016). However, few data are available on the role of virulence factors in *K. pneumoniae* pathogenicity. In this study, the epidemiological, clinical, and microbiological aspects of *K. pneumoniae*-related pyogenic liver abscesses were investigated in Shiraz. Although this is a monocentric study based on the situation of Abu Ali Sina Transplant Hospital (as the most important referral center for patients with liver diseases in Iran), the generalizability of the results to our country may be logical.

In this study, a significant change was observed in the etiologic aspect of PLAs. The prevalence of *K. pneumoniae* in pyogenic liver abscesses was 37% (20/54), which was higher than the prevalence of *E. coli* (30%, 16/54) as the most common pathogen in pyogenic liver abscesses, similar to previous reports (Rahimian et al., 2004; Chung et al., 2007). The higher prevalence of *K. pneumoniae* in PLAs reflects the presence of the hypervirulent pathotype of *K. pneumoniae*, which may explain why the incidence of *K. pneumoniae*-related pyogenic liver abscesses is increasing worldwide.

During our study period, the incidence of PLAs caused by hvKp was 4 (7%) of 54 cases of pyogenic liver abscesses, which is much lower than the cases reported in Southeast Asian countries, particularly Taiwan, China, and South Korea (Wang et al., 1998; Chung et al., 2007; Ye et al., 2016), and comparable to reports from Europe and North America (Anstey et al., 2010; Fazili et al., 2016; Rossi et al., 2018).

From a clinical perspective, hypervirulent *K. pneumoniae*-related pyogenic liver abscesses are usually monomicrobial and develop in individuals without underlying disease (Wang et al., 1998; Rossi et al., 2018). This is consistent with our study in which all four patients (100%) with hypervirulent *K. pneumoniae*-related pyogenic liver abscess were healthy and non-diabetic. In a report by Rossi *et al*. (Rossi et al., 2018), 50% of patients with hypervirulent *K. pneumoniae-*related pyogenic liver abscess were non-diabetic. Thus, in contrast to a previous report (Siu et al., 2012), diabetes does not appear to be a risk factor for hypervirulent *K. pneumoniae-*related pyogenic liver abscess. In our study, no metastatic infection with the source of PLA was found in patients with hypervirulent *K. pneumoniae-*related pyogenic liver abscess, whereas this phenomenon has been reported in previous studies (Fung et al., 2002; Tan et al., 2004; Sun et al., 2021). Diabetes is a known predisposing factor for impaired neutrophil chemotaxis and phagocytosis (Baras and Bin-Hameed, 2021). Patients with metastatic infections are usually diabetic, but some of them with metastatic infections by hvKp strains are healthy and have no history of diabetes, so another important factor may be involved in this phenomenon. In addition, hypervirulent *K. pneumoniae*-related pyogenic liver abscesses were frequently solitary and localized in the right lobe of the liver on medical imaging (3/4, 75%) (Siu et al., 2012).

Percutaneous needle aspiration or catheter drainage in combination with antibiotic therapy has been shown to be beneficial in the treatment of PLA (Justo et al., 2018). All patients in our study received antibiotics and underwent percutaneous aspiration or drainage. However, in agreement with previous reports, patients with hypervirulent *K. pneumoniae*-related pyogenic liver abscess had more favorable outcomes than patients with classic *K. pneumoniae*-related pyogenic liver abscess. This is due to the immune system of the patients. Because the patients with hypervirulent *K. pneumoniae*-related pyogenic liver abscess had no known impaired immunity (Siu et al., 2012; Rossi et al., 2018). When these therapeutic measures fail due to persistent abscess or sepsis, re-orthotopic liver transplantation (OLT) should be considered to avoid the high mortality associated with this severe complication (Justo et al., 2018). In the present study, the high mortality rate in cases with classic *K. pneumoniae*-related pyogenic liver abscess, 81% of whom were immunosuppressed, suggests that successful antibiotic therapy is highly dependent on an intact immune system.

Interestingly, *G. mellonella* larvae in the present study showed a significant difference in the virulence level of serotype K1, which was responsible for cryptogenic PLA, compared with serotype non-K1/K2, which was responsible for non-cryptogenic PLA. Dose- and time-dependent larval death was very pronounced in serotype non-K/K2 *K. pneumoniae* but not in serotype K1 *K. pneumoniae*, indicating the higher virulence of the hvKp strain compared with cKp (Zhao et al., 2019).

The results of our study show that in *K. pneumoniae* isolates, harboring any of the plasmid or chromosomal virulence genes contributes to the pathogenicity and high prevalence of *K. pneumoniae* isolates in PLA. In this regard, in the final phase of evolution, hypervirulent *K. pneumoniae* (hvKp) isolates with a specific genetic background are detected in cryptogenic invasive pyogenic liver abscesses. Because of the remarkable capability of *K. pneumoniae* to acquire mobile genetic elements (MGEs), including plasmids, transposons, and integrative conjugative elements (ICEs) (Lam et al., 2018b), we identified *K. pneumoniae* as the most common etiological agent of PLAs. We found a high prevalence of MDR classical *K. pneumoniae* (cKp) strains in non-cryptogenic pyogenic liver abscesses, and the presence of hvKp strains in cryptogenic pyogenic liver abscesses, which is concerning.

The main known virulence factors of *K. pneumoniae* include capsular polysaccharide (K antigen), which prevents phagocytosis by neutrophils and killing by serum complement, and siderophores, which bind ferric iron and are critical for bacterial growth and proliferation (Paczosa and Mecsas, 2016). Several siderophores are expressed in *K. pneumoniae*, including enterobactin, which is encoded by the chromosomal operon *entABCDEF* in all *K. pneumoniae* isolates, and yersiniabactin, which is encoded by the *ybt* locus within the integrative conjugative element of *K. pneumoniae* (ICE*Kp*). In our study, the *entB* gene was detected in all 20 K*. pneumoniae* isolates, consistent with previous reports (El Fertas-Aissani et al., 2013; Rossi et al., 2018). The *ybtS* gene was detected in all (100%) hvKp isolates and in 63% (10/16) of cKp isolates, indicating a high frequency of the *ybt* locus in cKp isolates, in contrast to a previous report (Paczosa and Mecsas, 2016). It appears that the presence of ICE*Kp* carrying the *ybt* locus in *K. pneumoniae* isolates contributes to the virulence and high prevalence of *K. pneumoniae* isolates in PLAs. Interestingly, in K1 ST23 hypervirulent *K. pneumoniae* strains, the *ybtS* gene was detected together with the *clbA* gene in ICE*Kp10*, as previous studies had shown (Struve et al., 2015; Lam et al., 2018a; Lam et al., 2018b).

Identification of a large virulence plasmid carrying aerobactin (*iucABCD-iutA*) and salmochelin (*iroBCDN*) siderophore gene clusters as well as *rmpA* (regulator of mucoid phenotype) and *rmpA2* genes in all hvKp strains, indicating the key role of this plasmid in the increased virulence of hvKp strains (Lee et al., 2017). In our study, K1/K2 hvKp strains associated with cryptogenic PLAs contained the siderophore operons aerobactin (*iucABCD-iutA*) and salmochelin (*iroBCDN*) as well as the *rmpA* and *rmpA2* genes. In addition, these strains formed colonies with a distinct hypermucoviscous phenotype on the blood agar plate, as described in previous reports (Ye et al., 2016; Rossi et al., 2018).

Potassium tellurite (K_2_TeO_3_) is an antibacterial agent. The toxicity of tellurite is due to its strong oxidizing ability (Taylor, 1999). However, tellurite resistance genes are detected on both plasmid and chromosome in several bacterial pathogens, including *Corynebacterium diphtheria*, *Staphylococcus aureus*, enterohemorrhagic *Escherichia coli* (EHEC), *Shigella* spp., *Yersinia pestis*, and *Bacillus anthracis*. Plasmid resistance is dependent on the *terZABCDEF* and *terW* gene cluster (Taylor, 1999; Passet and Brisse, 2015). In a report by Passte *et al*., 30.9% (38/123) of *K. pneumoniae* isolates from environmental sources or animal and human fecal samples, including hvKp strains belonging to clones ST23, ST65, and ST86, were resistant to tellurite and grew on MacConkey-inositol-potassium tellurite medium (MCIK) (Passet and Brisse, 2015). Interestingly, we found that 80% (16/20) of *K. pneumoniae* isolates, including all four hvKp strains belonging to clones ST23 and ST65, and 12 of 16 cKp strains were able to reduce tellurite to black metallic tellurium and form black colonies on tellurite-containing MH medium. The presence of tellurite resistance genes in most *K. pneumoniae* isolates from PLAs may contribute to the virulence and high prevalence of *K. pneumoniae* isolates in PLAs. The contribution of *ter* operon to the pathogenicity of *K. pneumoniae* isolates needs further investigation.

Liver tissue is the major storage site for iron, and it seems logical that a complete package of this large virulence plasmid in capsular serotypes K1/K2 is necessary for systemic liver affinity and the development of cryptogenic invasive pyogenic liver abscesses (Graham et al., 2007). Siderophore gene clusters, hypermucoviscosity genes, and tellurite resistance gene cluster induce the production of inflammatory cytokines TNF-α, IL-1β, and IL-6, as well as protection against phagocytosis and reactive oxygen species, and lead to intestinal tissue damage, systemic dissemination, and PLA formation (Fung et al., 2011).

When K1 hvKp and K2 strains were compared, three chromosomal regions of *kfu*, *allS*, and *clbA* were identified in all K1 hvKp strains but not in K2. The *kfu* gene mediates iron uptake, and the *allS* gene is involved in allantoin metabolism as a carbon and nitrogen source (Yu et al., 2008). The *clbA* gene is known to contribute to intestinal mucosal colonization, meningeal tropism, DNA damage, and brain cell death (Lu et al., 2017). In infections of mice, the NTUH-K2044 (Δ*allS*) mutant and the NTUH-K2044 (Δ*kfu*) mutant showed a significant decrease in virulence compared to the NTUHK-2044 wild-type strains (Chou et al., 2004; Ma et al., 2005). These three chromosomal regions likely contribute to virulence and a higher prevalence of capsular serotype K1. Interestingly, we also detected all three genes *kfu*, *allS*, and *clbA* in capsular serotypes K1 hvKp but not in K2, and a higher prevalence of K1 hvKp was observed.

Genotyping methods, including capsule typing and MLST, have shown that hvKp strains belong to restricted clones, whereas cKp strains are highly heterogeneous. The close clonal relationship of hvKp strains is particularly evident in capsular serotype K1, which belongs to clone ST23, the most common clone causing cryptogenic pyogenic liver abscesses. Clone ST23 not only represents the clonal distribution of capsular serotypes K1, but is a unique clone with exclusive genetic content. In contrast, capsular serotypes K2 are more genetically diverse and often belong to the clonal complexes CC65, CC86, CC375, and CC380. We also observed a clonal distribution of K1 hvKp strains belonging to ST23, while K2 hvKp strains belonged to ST65, which is consistent with previous reports (Bialek-Davenet et al., 2014; Luo et al., 2014; Rossi et al., 2018). Interestingly, we found that ST231 was the most common cKp strain responsible for three cases of non-cryptogenic PLA in three immunocompromised patients. Meanwhile, it was a co-producer of the carbapenemase OXA-48 and the extended-spectrum *β*-lactamase CTX-M. In addition to the *entB* gene, this strain also contained the *ybtS* and *kfu* genes, which may contribute to the virulence and higher prevalence of this cKp strain compared with other cKp strains. In a study by Shankar *et al.*, ST231 was the most common OXA48-like carbapenemase-producing *K. pneumoniae* in India (Shankar et al., 2019). Therefore, clone ST231 appears to be a high-risk clone with a high capacity for dissemination, colonization, pathogenicity, and exchange of virulence and antibiotic resistance genes.

In cKp isolates, mutations, efflux pumps overexpression, and enzyme production are mechanisms of antibiotic resistance. Consistent with our study, the production of ESBL and carbapenemase *β*-lactamases is the major mechanism of antibiotic resistance (Paczosa and Mecsas, 2016; Kareem et al., 2021). Unlike cKp isolates, which are often MDR, most hvKp strains reported to date are sensitive to antibiotics other than ampicillin (Lee et al., 2017), and this is consistent with our study. In hvKp isolates, an increased physical barrier of the capsule, plasmid incompatibility, and an active CRISPR/Cas system by reducing horizontal gene transfer (HGT) of mobile genetic elements may explain why these isolates are more sensitive to antibiotics than cKp isolates (Lam et al., 2018a). Nevertheless, the evolution of carbapenem-resistant hypervirulent *K. pneumoniae* strains has been frequently reported in Taiwan and China and poses a serious threat to public health (Gu et al., 2018; Lin et al., 2018).

A limitation of our study was the low prevalence of cases of hypervirulent *K. pneumoniae*-related pyogenic liver abscesses. This is likely due to the low rate of gastrointestinal carriers of hvKp strains. Intestinal carriage is a predisposing factor for hvKp liver abscess (Fung et al., 2012). Therefore, longer-term studies are needed to determine the identity of all hvKp strains.

Considering that hypervirulent *K. pneumoniae-*related pyogenic liver abscesses are emerging in Iran, physicians should be more cautious in diagnosing and treating patients with cryptogenic pyogenic liver abscesses. First, although no metastatic infections were observed in our study, invasion of hvKp strains from the liver to the eye, CNS, and other sites, which often occurs within the first three days of hospitalization, is usually devastating. Second, reports of antibiotic resistance in hvKp strains are increasing, and mortality rates in patients infected with these strains are high (Lee et al., 2008; Lin et al., 2018).

## Conclusions

In conclusion, this study demonstrates the evolution of opportunistic *K. pneumoniae* associated with non-cryptogenic PLAs in patients with underlying diseases to the true pathogen hvKp associated with cryptogenic invasive PLAs in healthy and young individuals in the community. The results of our study show that in *K. pneumoniae* isolates, the acquisition of any plasmid or chromosomal virulence genes contributes to pathogenicity and high prevalence in PLA. In this regard, hvKp isolates with a specific genetic background are responsible for cryptogenic PLAs. Meanwhile, the emergence of hvKp in PLAs may explain why the incidence of *K. pneumoniae*-related pyogenic liver abscesses is increasing worldwide. HvKp isolates are associated with a specific genetic background consisting of capsular serotypes K1/K2 harboring a large virulence plasmid carrying aerobactin (*iucABCD-iutA*) and salmochelin (*iroBCDN*) siderophore operons, as well as the *rmpA* (regulator of mucoid phenotype) and *rmpA2* genes. Interestingly, clone ST23 belonging to capsular serotypes K1 and clone ST65 belonging to capsular serotypes K2 are the most common clones of hvKp strains, respectively. HvKp isolates form colonies on the blood agar plate with a distinct hypermucoviscous phenotype not found in cKp isolates. We caution that hvKp poses a threat to human health because of its ability to cause invasive infections in young and healthy individuals.

## Data availability statement

The raw data supporting the conclusions of this article will be made available by the authors, without undue reservation.

## Ethics statement

The studies involving human participants were reviewed and approved by The studies involving human participants were reviewed and approved by the ethics committee of the Pasteur Institute of Iran (reference number: IR.PII. REC.1399.065). The patients provided their written informed consent to participate in this study. Written informed consent to participate in this study was provided by the participants’ legal guardian/next of kin.

## Author contributions

MS performed pus cultures, phenotypic testing, genotypic testing, and collected clinical and microbiological data. MS wrote the manuscript. MAN, MO, and AB wrote and revised the manuscript. AR, FM, and AA collected the samples. MS & ZH analyzed the data. FB & FS supervised the project and wrote and revised the manuscript. All authors read and approved the final manuscript.

## Funding

Funding was granted to Maryam Sohrabi as a Ph.D. student of the Pasteur Institute of Iran (Project No.: B-9636).

## Acknowledgments

The authors would like to thank the Biological Control Research Department, Plant Pathology Research Institute, Tehran, Iran, especially Dr. Jalal Shirazi, for providing the *Galleria mellonella* larvae. The authors would like to thank the staff of the Department of Bacteriology and Virology of Shiraz University of Medical Sciences, Abu Ali Sina Hospital, Namazi Hospital, Fara Parto Medical Imaging and Radiology Center, and Pasteur Institute of Iran. This research was supported by the Pasteur Institute of Iran (Project No.: B-9636).

## Conflict of interest

The authors declare that the research was conducted in the absence of any commercial or financial relationships that could be construed as a potential conflict of interest.

## Publisher’s note

All claims expressed in this article are solely those of the authors and do not necessarily represent those of their affiliated organizations, or those of the publisher, the editors and the reviewers. Any product that may be evaluated in this article, or claim that may be made by its manufacturer, is not guaranteed or endorsed by the publisher.
